# Editorial for the Special Issue on Emerging Micro Manufacturing Technologies and Applications, 2nd Edition

**DOI:** 10.3390/mi16080859

**Published:** 2025-07-25

**Authors:** Nikolaos Tapoglou

**Affiliations:** 1 Industrial Engineering and Management Department, International Hellenic University, 57001 Thessaloniki, Greece; ntapoglou@ihu.gr; 2 Laboratory of Machine Elements and Machine Design, School of Mechanical Engineering, Aristotle University of Thessaloniki, 54124 Thessaloniki, Greece

Manufacturing micro-components has become a key area of interest for research, owing to the growing demand for miniaturized components and assemblies, and a series of applications across multiple industrial sectors. The drive towards miniaturization is especially critical in industrial sectors like medical, consumer electronics, optics, and general engineering. These sectors drive the development of processes that can deliver components with sub-micron accuracy and flawless surface quality. Manufacturing processes like additive manufacturing, drawing, and electrochemical machining have been adapted to meet the requirements of microscale production, whilst on the other hand, processes like electrochemical deposition and electroforming have been developed with micromachining in mind. Research in the field, as in most manufacturing fields, has focused on the development of robust processes and simulation models that can lead to the optimization of micro manufacturing processes [1,2]. Building upon the success of the first edition of the *Emerging Micro Manufacturing Technologies and Applications* Special Issue [3], this second edition will continue to explore recent advances in the field. This issue features eight original research papers covering topics including micro-electronic device manufacturing, surface property modification, and additive manufacturing.

In particular, Liu et al. (Contribution 1) investigated the finishing process of the internal surface of cobalt chromium cardiovascular stents. Their research introduced a novel method for producing highly spherical Al_2_O_3_ magnetic abrasive particles that were used as an abrasive medium in the investigation. A dedicated experimental rig was produced for the magnetic abrasive finishing of long ultra-fine cardiovascular stents. Investigations were conducted on the influence of process parameters on the final stent quality. After processing, the inner surface roughness (Ra) of the tubes was reduced significantly from 0.337 µm to 0.09 µm, achieving a removal thickness of 5.106 µm.

Li et al. (Contribution 2) proposed a wire anode scanning electroforming method, which was used to improve the thickness uniformity of the electroformed metal layers. This process was based on a reciprocating paddle incorporating an ultra-fine inert wire anode. Numerical simulations allowed for the investigation of the process parameters and their effect on the deposited material characteristics. The experimental findings showcased an improvement in thickness uniformity by 15.5% on single-scale components and 11.4% on multi-scale component arrays, when compared to traditional electroforming.

Zuo et al. (Contribution 3) introduced a novel ultraviolet lithography-assisted sintering method for the fabrication of convex glass microlens arrays. The method involved the solidification of micropillar arrays of quartz glass using UV lithography, followed by debinding and sintering into the final shape. The fabrication method successfully demonstrated the manufacturing of lenses with diameters of 30, 40, 50, and 60 μm; MLAs with average focal lengths of 122, 151, 175, and 201 μm were used.

Cao et al. (Contribution 4) performed an experimental investigation into the drawing of single-crystal copper wires. They focused on the effect of drawing and annealing process parameters on the mechanical and electrical properties of the material whilst also taking into consideration the microstructure of the material. Experiments included multi-pass drawing with varying single-pass deformations and drawing speeds, followed by annealing at different temperatures and rates. The results showed that a 14% single-pass deformation led to optimal overall wire performance, increased tensile strength, and reduced resistivity. Drawing at 500 m/min increased tensile strength further. Optimal annealing temperatures were found to be 350–400 °C.

Yang et al. (Contribution 5) focused their investigation on a novel periodically reducing current (PRC) over-growth electroforming (EF) process. The process was used for fabricating ultra-narrow precision slits with widths of less than 10 μm. As part of their work, numerical simulations were coupled with experimental investigations. Numerical simulations revealed that the PRC mode significantly improved the cathode current density distribution and ion concentration within the narrowing slit. Experimental validation demonstrated that PRC over-growth EF can reliably achieve slits with a width of 5 ± 0.1 µm and a surface roughness of less than 62.8 nm.

Li et al. (Contribution 6) investigated the feasibility of fabricating microgroove structures on Zr-based metallic glass (MG) using jet electrochemical machining (jet-ECM) with sodium nitrate (NaNO_3_) electrolytes. The experimental investigation showed that Zr-based MG exhibits passivation, transpassivation, and re-passivation behaviors in NaNO3 solution. Based on the investigation, an applied voltage of 25 V, a nozzle travel rate of 100 μm/s, and a NaNO_3_ electrolyte concentration of 10 wt% yielded the optimal results in terms of accuracy and surface quality.

Zhang et al. (Contribution 7) proposed a periodically lifting necked entrance through the mask electroforming process. The goal of the investigation was the cost-effective fabrication of high aspect ratio micro- and mesoscale metallic components. Coupling simulation and experimental trials, the researchers demonstrated that an appropriate necked entrance size significantly improved the flow field and current density uniformity.

Finally, Huang et al. (Contribution 8) developed a scalable DLP additive manufacturing method to simultaneously print large and small features with high local shape accuracy. The method achieved minimum printing resolutions of 101 µm (at 20.5 cm) and 157 µm (at 30.5 cm). When compared to traditional DLP, the proposed method offered an improved accuracy of small features, reducing both roundness and straightness errors.
